# Rifamycin O, An Alternative Anti-*Mycobacterium abscessus* Agent

**DOI:** 10.3390/molecules25071597

**Published:** 2020-03-31

**Authors:** Bui Thi Bich Hanh, June-Woo Park, Tae Ho Kim, Jae-Sung Kim, Chul-Su Yang, Kiseok Jang, Jinsheng Cui, Dong-Chan Oh, Jichan Jang

**Affiliations:** 1 Molecular Mechanisms of Antibiotics, Division of Life Science, Research Institute of Life Science, Gyeongsang National University, Jinju 52828, Korea; hanhm0515006@gstudent.ctu.edu.vn (B.T.B.H.); taeho12349@gmail.com (T.H.K.); 2 Division of Applied Life Science (BK21plus Program), Gyeongsang National University, Jinju 52828, Korea; 3 Environmental Biology Research Group, Korea Institute of Toxicology, Jinju 52834, Korea; jwpark@kitox.re.kr; 4 Human and Environmental Toxicology Program, Korea University of Science and Technology (UST), Daejeon 34113, Korea; 5 Department of Bionano Technology, Hanyang University, Seoul 04763, Korea; sung901017@naver.com (J.-S.K.); chulsuyang@hanyang.ac.kr (C.-S.Y.); 6 Department of Molecular & Life Science, Hanyang University, Ansan 15588, Korea; 7 Department of Pathology, Hanyang University College of Medicine, Seoul 04763, Korea; medartisan@hanyang.ac.kr; 8 Natural Products Research Institute, College of Pharmacy, Seoul National University, Seoul 08826, Korea; cuijs@snu.ac.kr (J.C.); dongchanoh@snu.ac.kr (D.-C.O.)

**Keywords:** rifamycin, *Mycobacterium abscessus*, zebrafish bacterial infection, drug resistance, non-tuberculous mycobacteria

## Abstract

*Mycobacterium abscessus* is the most difficult-to-treat nontuberculous mycobacteria because of its resistance to many antibiotics. In this study, we screened the Korea Chemical Bank library for a bioluminescent reporter assay to identify molecules capable of acting against *M. abscessus.* On application of the assay, rifamycin O showed excellent in vitro activity with a narrow range of the minimum inhibitory concentration required to inhibit the growth of 90% of the bacterium (MIC_90_ = 4.0–6.2 μM); its in vivo efficacy in the zebrafish (*Danio rerio*) infection model was comparable to that of rifabutin at 25 μM. Furthermore, rifamycin O did not show significant toxicity in cells and the zebrafish model. These results are the first in vivo indication that rifamycin O may be a drug candidate for treating *M. abscessus* infections.

## 1. Introduction

*Mycobacterium abscessus* is a rapidly growing saprophyte, commonly found in soil and water [1]. *M. abscessus* is known to cause chronic lung and skin infections in immunocompromised hosts, which are difficult to treat due to antimicrobial drug resistance [2]. In order to treat the infections caused by *M. abscessus*, a multi-drug cocktail comprising clarithromycin, amikacin, and cefoxitin or imipenem has been used [3]. Clarithromycin is known to be the most effective drug used to treat *M. abscessus* [3]. However, there is still a high rate of treatment failures (20%–73%) because *M. abscessus* has both natural and acquired drug resistance to clarithromycin [4]. Thus, there is a pressing need for alternative drugs that could replace clarithromycin. Based on whole-genome studies, *M. abscessus* comprises three different subspecies: *M. abscessus* subsp. *abscessus*, *M. abscessus* subsp. *bolletii*, and *M. abscessus* subsp. *massiliense* [5]. Interestingly, the three subspecies show different resistance profiles to clarithromycin. For example, *M. abscessus* subsp. *massiliense* is less resistant to clarithromycin than the other two subspecies, because it contains a non-functional *erm(41)* gene that confers inducible macrolide resistance. In contrast, *M. abscessus* subsp. *abscessus* and *M. abscessus* subsp. *bolletii* have an intact *erm(41)* gene that provides clarithromycin resistance. However, the *M. abscessus* subsp. *abscessus* MAB30 subgroup has a T to C substitution at position 28 of *erm*(T28C), which consequently enables its sensitivity to clarithromycin [6].

Rifamycin is an RNA polymerase-targeting antimicrobial agent, and rifampicin is a well-known rifamycin analog. Currently, rifampicin is used to treat tuberculosis and leprosy [7]. Rifampicin binds to the β-subunit (RpoB) of the DNA-dependent RNA polymerase in prokaryotes, and consequently inhibits bacterial transcription. However, rifampicin is not an effective agent against *M. abscessus* because *M. abscessus* contains target-modifying enzymes such as intrinsic ADP-ribosyltransferase (MAB_0591 gene product Arr_*Mab*) that mediate rifamycin resistance. Based on the findings reported by Rominski et al., the genetic approach with the Arr_*Mab*-deletion mutant showed much lower minimum inhibitory concentration (MIC) values than the parental strain, and its compensated strain restored the wild type phenotype against rifamycin [8]. Thus, rifampicin is not useful for treating *M. abscessus* infections. However, recently, Aziz et al. reported that rifabutin, another rifampicin analog, showed excellent in vitro activity against the *M. abscessus* reference strain, three *M. abscessus* subspecies, and clinical isolates in comparison with other rifamycin analogs such as rifampicin and rifapentine. Furthermore, a recent study reported that rifabutin had as good therapeutic effects against *M. abscessus* K21 strain in NOD/SCID (Nonobese Diabetic/Severe Combined Immunodeficiency) mice as clarithromycin [9]. Therefore, an increased understanding of the rifamycin analog mechanism of action via a structure-based study would be highly desirable.

This study aimed to search for alternative compounds for treatment of *M. abscessus* infections by screening all available compounds deposited in the Korea Chemical Bank (KCB) library. Screening was conducted using a bioluminescent *M. abscessus*-based assay, and MIC values of the hits were generated by resazurin microtiter assay. The sorted optimal compound was rifamycin O, its activity was subsequently evaluated against different strains and subspecies of *M. abscessus* either in vitro or in vivo using zebrafish (ZF) as a model of infection.

## 2. Results

### 2.1. Identifying Active Compounds against Bioluminescent M. abscessus from a KCB Library

**Figure 1:**
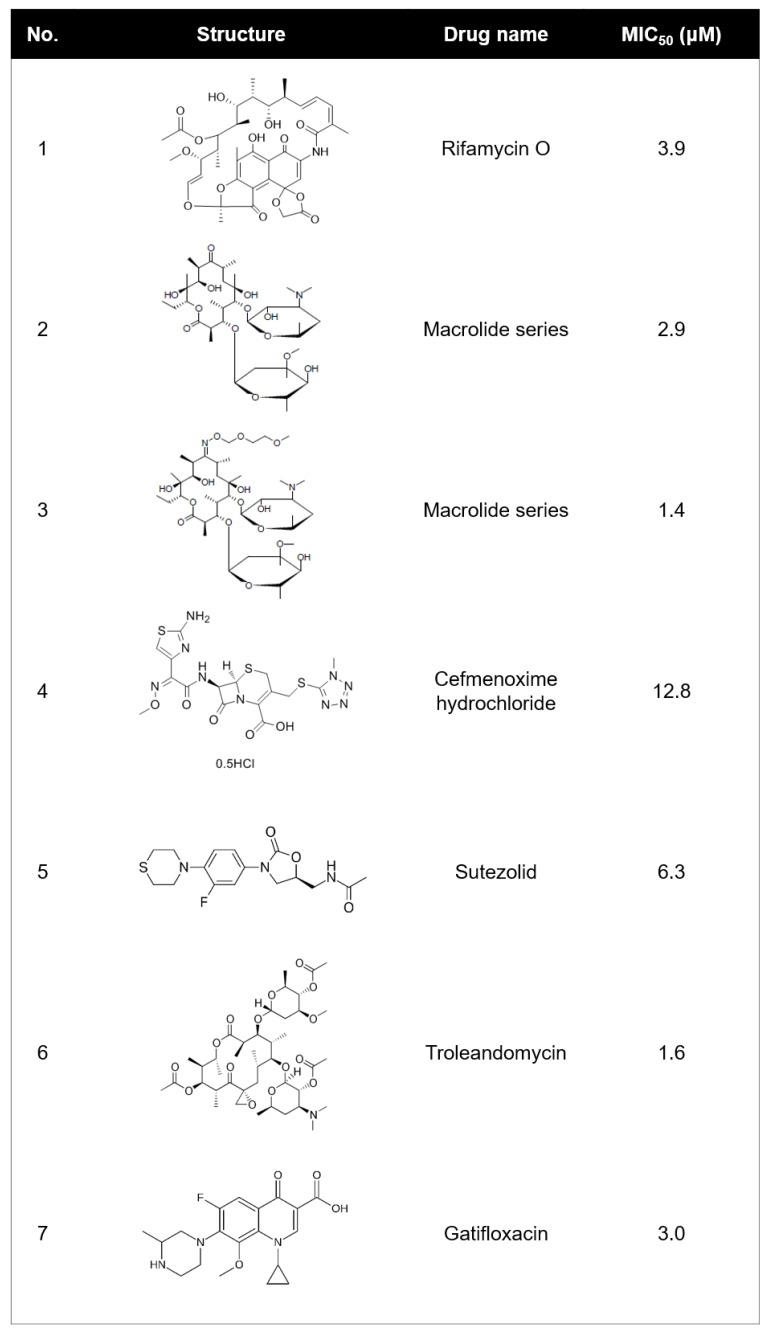

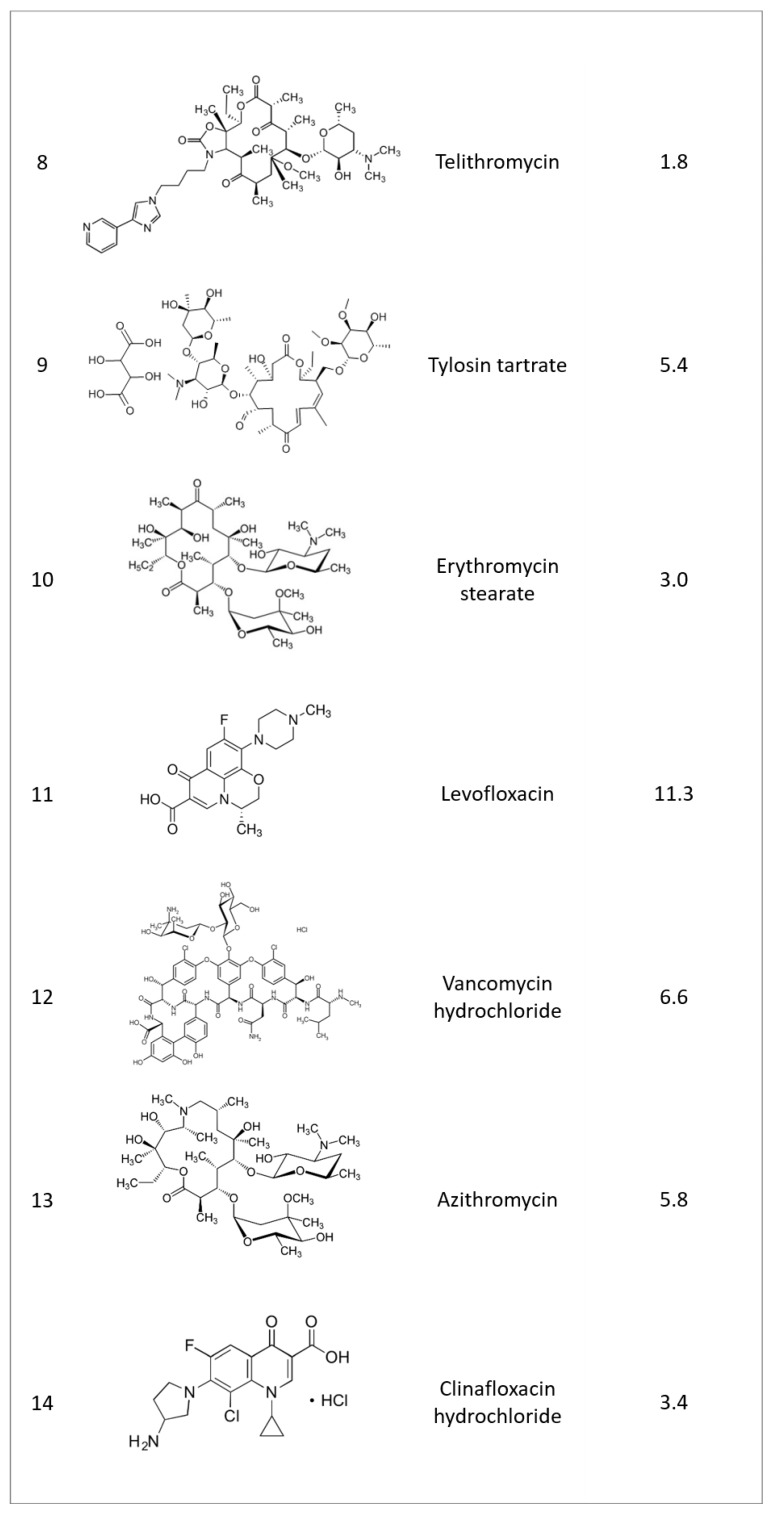

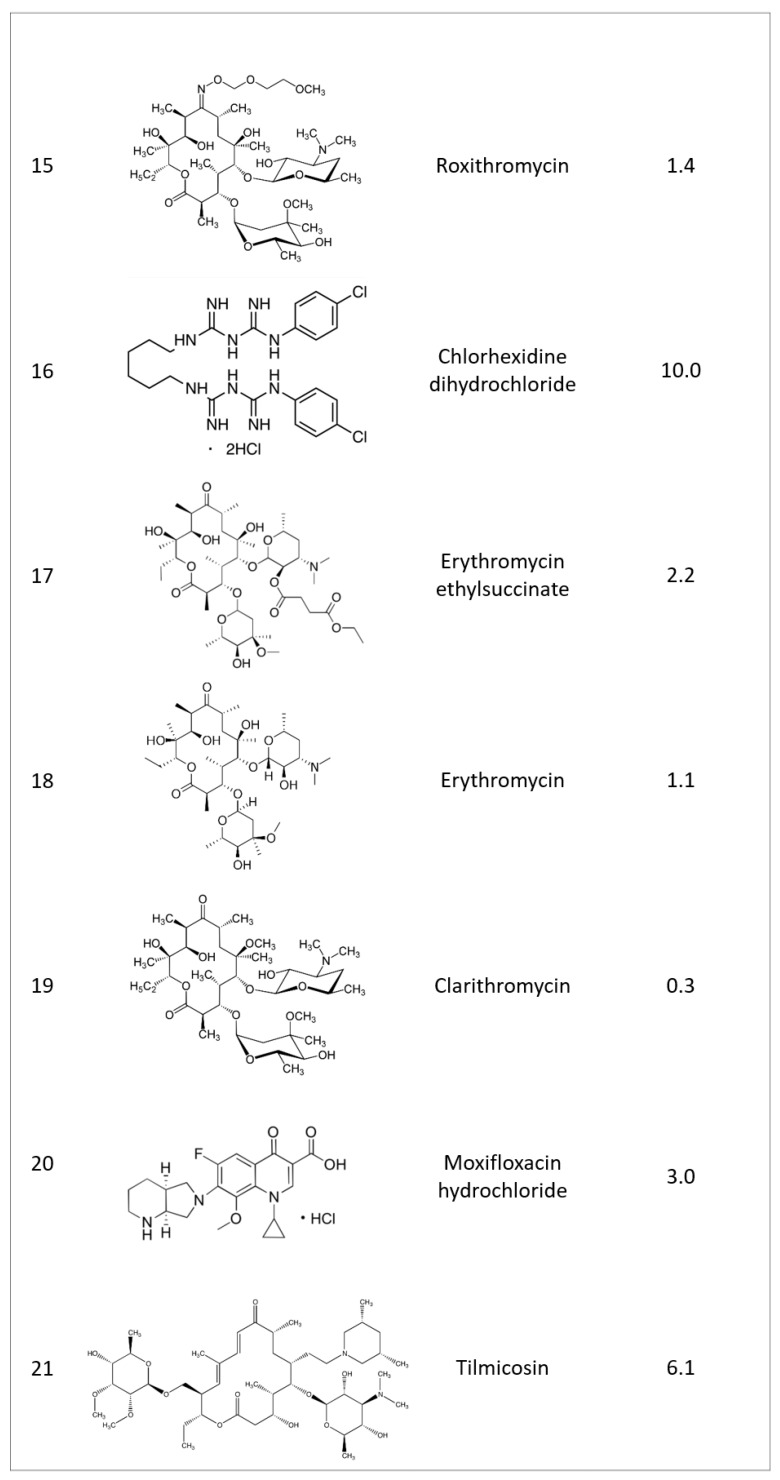

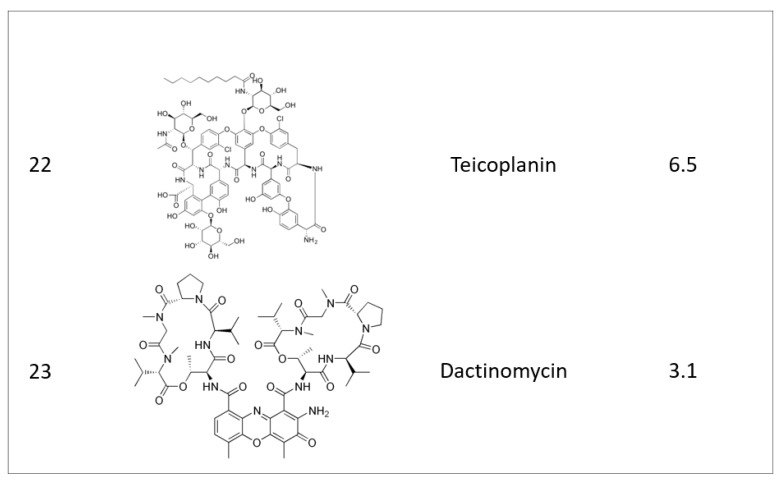
Structure and 50% minimum inhibitory concentration (MIC_50_) values of the 23 most potent *M. abscessus* hits.

Compounds with an MIC_50_ of ≤15 μM were selected as being active in whole-cell; 23 active compounds were identified (0.17% “hit” rate). Their structures and activities are shown in Figure 1. Most of the candidates were already-known antibiotics, such as those from the macrolide and fluoroquinolone families. However, we found that rifamycin O that had an MIC_50_ of 3.9 μM against bioluminescent *M. abscessus*. This discovery was very interesting, because structurally similar rifamycin analogs have been poorly active to *M. abscessus*. For this reason, the rifamycin family has not been considered as a therapeutic option to treat *M. abscessus* infection. However, recently, rifabutin has been identified as a repurposing candidate that has an activity for in vitro *M. abscessus*, and mouse models [9,10]. This rifabutin has different chemical structures compared with other rifamycin analogs. It lacks a hydroquinone, which is considered as a key factor for anti-*M. abscessus* activity at the C1 and C4 positions [11]. Interestingly, rifamycin O shows a structural similarity with rifabutin, especially the absence of hydroquinone. Therefore, we characterized the activity of rifamycin O against *M. abscessus* either in vitro or in vivo using zebrafish (ZF) to highlight the importance of the absence of hydroquinone in rifamycin to treat *M. abscessus*.

### 2.2. Rifamycin O is Non-toxic to Cells and Inhibits In Vitro Growth of M. abscessus CIP 104536 ^T^

**Figure 2:**
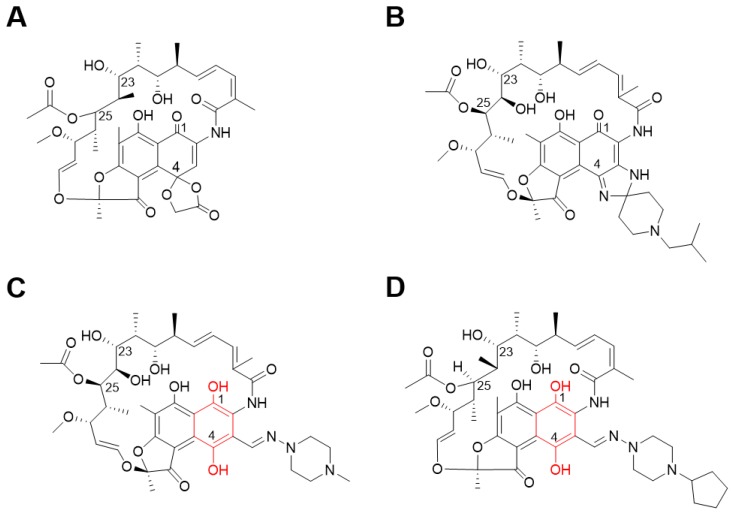
Chemical structures of rifamycin analogs. Rifamycin O (**A**), rifabutin (**B**), rifampicin (**C**), and rifapentine (**D**). Red indicates hydroquinone.

The isolated rifamycin analog was re-purchased from PHARMEKS (cat. #PHAR087207). The structure of the rifamycin analog was confirmed as rifamycin O by LC/MS and 1D and 2D NMR data (Figure 2A). The cytotoxic effect of rifamycin O was examined in mouse bone marrow-derived macrophages (mBMDM). As shown in Figure S1, rifamycin O was non-cytotoxic to cell cultures at concentrations up to 100 μM (Figure S1).

**Figure 3:**
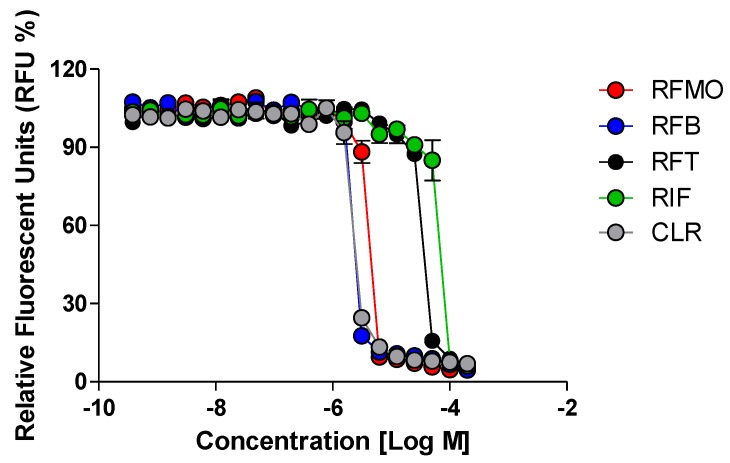
In vitro activity of rifamycin O. The activity of rifamycin O (RFM O) against *M. abscessus* CIP 104536^T^ R morphotype in comparison with rifabutin (RFB), rifampicin (RIF), rifapentine (RFT) and clarithromycin (CLR) in 7H9^G/T/ADC^. Fluorometric minimum inhibitory concentrations (MICs) were determined by fitting the RFU% sigmoidal dose–response curves. Graph fitting is representative of three independent assays, performed in triplicate.

To test whether rifamycin O has a growth-inhibitory effect on *M. abscessus*, we conducted a resazurin-based drug-susceptibility test with *M. abscessus* CIP 104536^T^ R in 7H9^G/T/ADC^ with reference compounds such as rifapentine, rifabutin, rifampicin, and clarithromycin. As seen in Figure 3, rifamycin O significantly decreased resazurin fluorescence in a concentration-dependent manner. The rifamycin O showed strong inhibitory activity, with an MIC_50_ as low as 3.9 μM. The positive controls (rifabutin and clarithromycin) also showed a very strong anti *M. abscessus* activity. In contrast, rifampicin and rifapentine showed very high MIC_50_ (> 20 μM), as described previously [10].

Furthermore, we compared the MIC_90_ of rifamycin O with that of rifapentine, rifabutin, rifampicin, and clarithromycin against three different *M. abscessus* subspecies. As shown in Table 1, rifamycin O showed much stronger activity than rifampicin and rifapentine, and showed similar activity to rifabutin and clarithromycin. Rifamycin O showed strong inhibitory activity, with a low MIC_90_ of 4.0-6.2 μM, against *M. abscessus* subsp. *bolletii* CIP108541^T^, *M. abscessus* subsp. *massiliense* CIP108297^T^ and *M. abscessus* CIP 104536^T^ S. These results suggest that rifamycin O is an effective compound against all phylogenetically close *M. abscessus* subspecies.

### 2.3. In Vivo Rifamycin O Efficacy Assessment Using ZF Embryo

**Figure 4:**
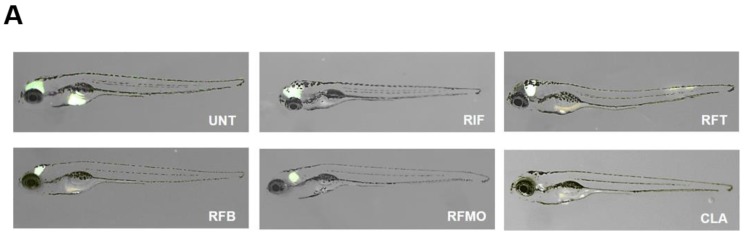

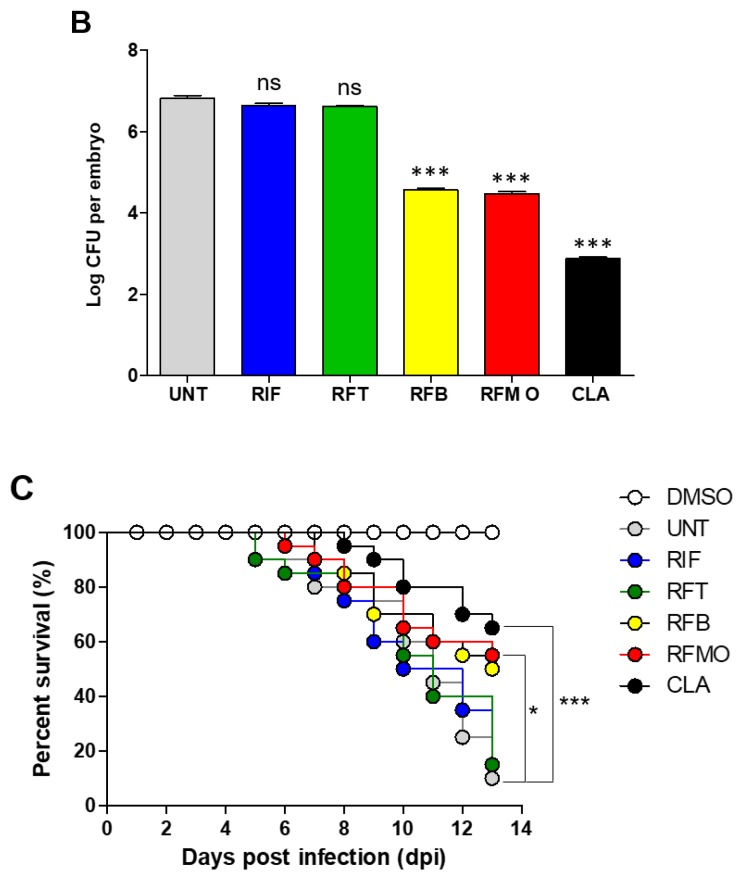
Evaluation of in vivo RFM O activity on *M. abscessus* CIP 104536 R morphotype expressing mWasabi infection. Zebrafish (ZF) were infected with *M. abscessus* CIP 104536^T^ R expressing mWasabi, and treated with different antibiotics. The *M. abscessus* infected ZF were exposed to RIF, RFT, RFM O, RFB and CLR at a concentration of 25 μM and compared to untreated controls. Green fluorescent protein (GFP) dissemination in ZF was captured using fluorescent microscopy (**A**). The 5 dpi embryos at 25 µM of RIF, RFT, RFM O, RFB and CLR show significant reductions in infection burden (*** *P* < 0.0001; ns, not significant) (**B**). Survival of *M. abscessus*-infected embryos treated at 25 μM of rifamycin analogs and clarithromycin (**C**) in comparison with untreated infected embryos and non-treated control (*n* = 20, representative of three independent experiments). * P < 0.05, *** P < 0.001.

The in vivo efficacy was tested in a ZF model of infection using *M. abscessus* CIP 104536^T^ R-type. Initially, the maximum-tolerated rifamycin O dose (MTD) was determined using an escalating dose of rifamycin O in ZF. To do this, non-*M. abscessus* infected ZF were treated with 5, 10, and 25 μM of rifamycin O. As shown in Figure S2, rifamycin O-treated ZF did not show reduced survival rates, until 7 days after treatment. However, on day 8, 60%–74% of the ZF died across all doses used. Furthermore, 100% of the ZF died 9 days after rifamycin O treatment. The survival curve was compared with that of two different rifamycin analogs, rifabutin and rifampicin, that have previously been used in clinic as control. In the test, rifabutin and rifampicin (dose at 5, 10, and 25 μM) also showed similar toxicity in ZF. In this case, 47%–65% of the fish died and 70%–72% of the fish died on day 8 after treatment with rifabutin and rifampicin, respectively. By day 9, no ZF had survived. Based on this, we concluded that all three concentrations of rifamycin O, rifabutin, and rifampicin show similar toxicity profiles in ZF and that 25 μM of each compound could be used for an in vivo efficacy study. Based on this compound-toxicity result, we injected ~40 CFU of *M. abscessus* into ZF and treated ZF with 25 μM of rifampicin, rifapentine, rifabutin, and rifamycin, and with clarithromycin as a positive control, to evaluate the survival of *M. abscessus* in ZF in an vivo-treatment model. The *M. abscessus* dissemination in ZF was observed under a fluorescence microscope using virulent *M. abscessus* CIP 104536^T^ R expressing GFP. After bacterial infection, each rifamycin analog was administered at a dose of 25 μM daily up to 5 dpi. As shown in Figure 4A, infected *M. abscessus*-GFP was gradually disseminated in the untreated control. The amount of GFP increased in ZF yolk and head at 5 dpi. However, only a limited and small amount of GFP expression was observed in the head region when rifamycin O was administered to *M. abscessus*-GFP infected fish at 5 dpi. A similar GFP reduction was also observed with the 25 μM rifabutin treatment. However, rifampicin- and rifapentine- treated fish showed comparatively much larger GFP-expressing area, compared to fish treated with rifabutin and rifamycin O. Fish treated with the clarithromycin positive control displayed only a tiny amount of GFP at 5 dpi.

This GFP dissemination was double-validated using CFU enumeration to confirm the reduction of the live bacterial burden inside *M. abscessus*-infected ZF after compound treatment. After treatment with 25 μM of rifamycin O, rifabutin, rifampicin and rifapentine, individual ZF were homogenized and their CFUs were quantified. As displayed in Figure 4B, the untreated negative control showed a maximum live bacterial burden at 5 dpi. However, rifamycin O showed excellent live-bacterial CFU reduction at the 25 μM concentration. This effectiveness was similar to that of the rifabutin treatment at 25 μM. In contrast, rifampicin and rifapentine failed to reduce the bacterial burden inside the *M. abscessus*-infected ZF. Taken together, these results suggest that both Rifamycin O and rifabutin have a therapeutic effect against *M. abscessus*; such an effect is not seen with rifampicin and rifapentine in vivo.

Furthermore, we determined the percentage of survival by generating a Kaplan–Meier survival curve. Figure 4C shows that, nearly 100% of non-*M. abscessus* infected ZF can survive until day 13. In contrast, 90% of *M. abscessus*-infected ZF that did not receive treatment had died by day 13. However, fish treated with 25 μM of rifamycin O showed significantly extended lifespans, with 54% of *M. abscessus* infected ZF surviving up to 13 days. This indicates that rifamycin O, at a dosage of 25 μM, is an efficient compound for treating *M. abscessus* infection in ZF. Both rifabutin and clarithromycin positive controls showed similar survival-rate profiles to the rifamycin O treatment; 50% and 65%, respectively.

**Figure 5:**
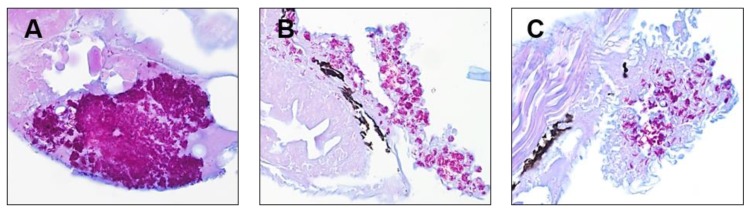
Multiple acid-fast bacilli are present in the ZF. ZF with *M. abscessus* CIP 104536^T^ R morphotype histopathological analysis was performed by Ziehl–Neelsen (acid-fast) staining. Non-treated *M. abscessus* CIP 104536^T^ R-infected ZF (**A**). *M. abscessus* CIP 104536^T^ R-infected ZF treated with RFM O (**B**) and CLR (**C**). Magnification, ×400.

We further evaluated whether rifamycin O could reduce proliferation of the *M. abscessus* CIP 104536^T^ R-type strain in ZF. Sections of ZF were examined for the presence of *M. abscessus* using Ziehl–Neelsen (AFB) staining. As shown in Figure 5A, untreated *M. abscessus*-infected ZF exhibited a high bacterial burden inside ZF. On the contrary, relatively few acid-fast bacilli were detected in specimens from ZF treated with rifamycin O (Figure 5B). Additionally, the clarithromycin-control experiment showed an excellent reduction of bacterial content (Figure 5C). These results illustrate the efficacy of rifamycin O in an in vivo model of bacterial infection.

## 3. Discussion

Because *M. abscessus* has intrinsic and acquired resistance mechanisms and *M. abscessus* cell wall is 10–20 times less permeable than *M. tuberculosis*, *M. abscessus* is more resistant to many antibiotics, including anti-tuberculous agents. Thus, anti-tuberculosis drugs have not been used to treat *M. abscessus* infected patients in the clinic. The generation of new active compounds against *M. abscessus* is urgently needed [12,13]. In this study, we screened 13,840 KBC compounds and identified rifamycin O as showing significant activity against the *M. abscessus* complex in vitro. Rifamycin O (4-*O*-(carboxymethyl)-1-deoxy-1,4-dihydro-4-hydroxy1-oxorifamycin *γ*-lactone) is an antibiotic rifamycin derivative obtained by the oxidation of natural rifamycin B, produced from *Streptomyces mediterranei* [13]. Although most of rifamycin resistance is caused by mutations in *rpoB*, *M. abscessus* and *Mycobacterium smegmatis* have an additional rifamycin-activating mechanism that uses rifampicin ADP-ribosyltransferase (Arr enzymes). The Arr enzyme of *M. abscessus* (Arr_*Mab*) also inactivates rifamycin and its derivatives, through the addition of an ADP-ribosyl group to the C23 hydroxyl group of the rifamycin [14]. However, recently, Aziz et al. reported an in vitro application of rifabutin, a close analog of rifamycin, against *M. abscessus* reference strains and clinical isolates [10]. This suggests that a rifamycin analog can escape Arr_*Mab*-mediated rifamycin-resistance mechanisms. Previously, some reports noted the importance of the replacement of the C25 acetate of rifamycin with carbamate for improving activity against *M. smegmatis* and against rapidly growing pathogenic mycobacteria. Relatively large groups at the position C25 of rifamycin, such as a carbamate, effectively block ribosylation of the C23 alcohol by Arr_*Mab* [15,16]. However, rifamycin O is not structurally different from other rifamycin analogs at position C25. Additionally, rifamycin O does not differ significantly from the general structure of other rifamycin analogs (Figure 2A). However, rifamycin O does exhibit differences from other rifamycin’s, mainly at the C1 and C4 positions. Many rifamycin analogs have been synthesized, but currently only rifabutin has been found to be effective against *M. abscessus*. The uniqueness of rifabutin can be explained by the structural differences between rifampicin and rifapentine at the C1 and C4 positions. Rifampicin contains hydroquinone which can easily be oxidized into rifampicin quinone in the presence of oxygen and divalent cations. However, the rifabutin does not contain hydroquinone. Rifabutin’s lack of hydroquinone confers resistance to autoxidation and consequently retains its activity against *M. abscessus* under oxidizing conditions (Figure 2B) [11]. Interestingly, both rifamycin O and rifabutin lack hydroquinone in the same positions; rifamycin O consequently showed very similar activity to rifabutin. Thus, we speculate that this structural uniqueness at the C1 and C4 positions is responsible for the activity of rifamycin O against *M. abscessus*. Pharmacophore-based virtual screening and molecular modeling studies based on the structure–activity relationship of rifamycin O by medicinal chemists will likely explain this structural uniqueness in the near future.

Most immunocompetent mouse strains such as C57BL/6 are resistant to *M. abscessus*, and eventually result in clearance of *M. abscessus* in the first weeks after infection [12]. Thus, this has created a bottleneck in evaluating the therapeutic efficacy of potentially effective antibiotics against *M. abscessus* in animal models. However, a potential mouse model was recently developed to produce a progressive high level of infection with *M. abscessus*. It is based on mice with certain immune defects such as SCID (severe combined immunodeficiency), gamma interferon knockout (GKO), and granulocyte-macrophage colony-stimulating factor (GMCSF) knockout mice [15,17]. Furthermore, recently, Thomas et al. successively reported the efficacy of rifabutin against *M. abscessus* K21 strain in NOD SCID mice, which have impaired B and T lymphocytes and diminished natural killer cell function [9]. However, although the immunocompromised mice showed a significant advancement in mouse models assessing drug efficacy against *M. abscessus*, the use of immunocompromised mice is relatively costly and time consuming. Furthermore, it may not reflect the predictive value required for compound testing [12].

In this aspect, ZF and ZF embryos have become a popular alternative to study therapeutic efficacy against *M. abscessus*. ZF has a very similar cellular composition in innate immunity to humans, and their optical transparency allows non-invasive real-time monitoring of bacterial infection and host–pathogen interactions [18]. Furthermore, it is relatively simple and cost-effective [19]. Nonetheless, the ZF model has some obvious limitations when compared to mammalian models. For example, ZF have gills instead of lungs and a lack of adaptive immunity in early development. Therefore, ZF embryos are more suited to studying acute infection rather than chronic disease, with *M. abscessus* [12].

Utilizing an in vivo ZF-infection model, we injected *M. abscessus* CIP 104536^T^ R via the caudal vein into early embryos and initiated treatment with rifamycin O and reference compounds to confirm rifamycin O activity against early *M. abscessus* infection. Microinjection of *M. abscessus* CIP 104536^T^ R resulted in the death of nearly all infected ZF by 13 dpi, while treatment with rifamycin O resulted in a significantly enhanced lifespan of up to 13 days (~54% survival rate); this result was similar to the treatment with rifabutin (~50% survival rate). Furthermore, rifamycin O treatment showed excellent bacterial reduction in CFU quantification, and Ziehl–Neelsen staining. Based on these results, we concluded that rifamycin O shows promise as an in vivo anti-*M. abscessus* compound. However, we have yet to test rifamycin O activity against infection in a higher animal model. Testing the in vivo efficacy of rifamycin O in immunocompromised mice will be a viable and worthy study to conduct in future.

In our study, we demonstrated the activity of rifamycin O in vitro and in vivo in ZF. Therefore, rifamycin O may be considered a possible drug candidate for the treatment of *M. abscessus* infections.

## 4. Materials and Methods

### 4.1. Ethics

All ZF experiments were approved by the animal-research ethics committee of Gyeongsang National University (GNU-190325-E0014).

### 4.2. Bacterial Strains and Growing Conditions

*Mycobacterium abscessus* subsp. *abscessus* CIP 104536^T^ smooth (S)- and rough (R)-morphotypes were provided by Laurent Kremer (CNRS, IRIM, Universite’ de Montpellier, Montpellier, France). *Mycobacterium abscessus* subsp. *bolletii* CIP108541^T^ and *Mycobacterium abscessus* subsp. *massiliense* CIP108297^T^ were obtained from the Collection de l’Institut Pasteur (CIP, Paris, France). Clinical isolates were purchased from the Korea Mycobacterium Resource Center (KMRC, Osong, Korea). The bioluminescent *M. abscessus* CIP 104536^T^ S strain (hereafter bioluminescent *M. abscessus*), that harbors the bacterial lux operon using pMV306hsp+LuxG13 (Addgene #26161, Cambridge, MA, USA) was constructed as previously described [20]. Any *M. abscessus* strains were grown at 37 °C either in liquid or solid media. Middlebrook 7H9 broth was used as the liquid medium, which contained 0.2% glycerol (*v*/*v*), 0.05% Tween 80 (*v*/*v*) and was supplemented with albumin-dextrose-catalase (ADC) (*v*/*v*) (7H9^G/T/ADC^), while the solid medium was Middlebrook 7H10 agar that contained 0.05% Tween 80 (*v*/*v*) supplemented with 10% oleic acid-ADC (OADC) (*v*/*v*) (7H10^T/OADC^). Reagents and media compositions were purchased from Sigma-Aldrich (St. Louis, MO, USA). Recombinant *M. abscessus* CIP 104536^T^ R morphotype carrying a pMV262-GFP plasmid that express green fluorescent protein (GFP) was prepared as previously described [21]. To prepare fresh injection stock, *M. abscessus* (∼5 × 10^5^ colony-forming unit (CFU)) expressing GFP was harvested by centrifugation at 4000× *g* for 5 min, and the pellet was washed twice using 100 μL of 7H9^G/T/ADC^. The pellet was resuspended in 200 µL 7H9^G/T/ADC^ and aliquoted into volumes of 5 µL into PCR tubes and stored at −80 °C. Prior to injection, the CFU level of the inoculum was verified by plating out serial dilutions. For the infection, the bacteria inoculum was diluted with PBST (Phosphate-Buffered Saline with 0.05% Tween 80) and resuspended in Phenol Red 0.085% to obtain 130 CFU/nL.

### 4.3. Bioluminescent Reporter-Based Screening and Determination of Minimum Inhibitory Concentrations

The assay was validated using the Z-factor as previously described [22]. A Chemical Library (13,840 compounds) was provided by the Korea Chemical Bank (KCB, Daejeon, Korea), preformatted in master plates so that all compounds were solubilized in 100% Dimethyl Sulfoxide (DMSO) at a final concentration of 2 mM. A Biomek 4000 Automated Liquid Handler (Beckman Coulter, Fullerton, CA, USA) was used for all liquid handling protocols. We screened the compounds against the bioluminescent *M. abscessus*. Bacterial culture with a final inoculum of 5 × 10^5^ CFU/mL in 7H9^G/T/ADC^ dispensed into 96-well flat clear bottom white microplates (99 µL per well) (Corning, Baltimore, MD, USA) and 1 µL of compounds were added to each well in a final drug concentration of 20 μM. A bacteria combination with DMSO at 1% final concentration (*v*/*v*) and bacteria in combination with 1 μL of 1 mM clarithromycin (Sigma) were included on each assay plate as positive and negative controls, respectively. The plate was incubated for 40 h at 37 °C and sample luminescence was measured with a SpectraMax^®^ M3 Multi-Mode Microplate Reader (Molecular Devices, San Jose, CA, USA).

The MIC_50_ values of the hit compounds were determined using the resazurin microtiter assay (REMA). Briefly, 100 μL of 7H9^G/T/ADC^ was added to every well of a 96-well microtiter plate and two-fold serial dilutions of hit compounds were prepared. *M. abscessus* was diluted to a final inoculum of 5 × 10^5^ CFU /mL in wells of a 96-well microtiter plate. The plates were covered and incubated at 37 °C for 3 days. Then, 20 μL of 0.025% (*w*/*v*) resazurin was added to each well and the plates were re-incubated overnight [23]. Fluorescence was measured (ex. 560/em. 590 nm) using the microplate reader. A dose–response curve was plotted using Prism 6 (GraphPad Software, Inc., La Jolla, CA, USA) to generate a minimum inhibitory concentration that inhibits 50% of the test bacterium (MIC_50_).

### 4.4. Chemical Analysis

Low-resolution mass-spectrometric data were acquired using an Agilent Technologies 6130 Quadrupole mass spectrometer (ESI: Electrospray Ionization, Santa Clara, CA, USA) coupled with an Agilent Technologies 1200 series HPLC (High Performance Liquid Chromatography) (HPLC column: Phenomenex reversed-phase C_18_(2), 5 μm, 4.6 × 100 mm, gradient solvent conditions: 10%–100% CH_3_CN/H2O over 20 min, 100% CH_3_CN after 20 min, flow rate: 0.7 mL/min). Rifamycin O was detected at 18.0 min. ^1^H, ^13^C, and HSQC (Heteronuclear Single Quantum Coherence), COSY (COrrelation SpectroscopY), and HMBC (Heteronuclear Multiple-Bond Connectivity) NMR experiments for riframycin O were performed using a Bruker Avance 800 MHz spectrometer (Billerica, MA, USA). Analysis of the 1D and 2D NMR spectra along with the mass spectrometric data confirmed the structure of rifamycin O.

### 4.5. Cytotoxicity Assay

For the bone marrow-derived macrophage (BMDM) cell-viability assay, a Quanti-Max WST-8 Cell Viability assay kit (BIOMAX, Seoul, Korea) was used according to the manufacturer’s instructions. Cells were placed into a 96-well plate (1.0 × 10^5^ cells / well) and incubated at 37 °C for 24 h. Various concentrations of hits were then added to the cells. After an additional 24 h incubation period, 10 μL of reagent (10% media volume) was added to each well, and the incubation was continued for 4 h. The resulting color was assayed at 450 nm using the microplate reader. BMDM cells lysed by 1% Triton-X-100 were used as the positive control, while the 1% DMSO treated BMDM cells were assayed as a negative control.

### 4.6. Drug Efficacy Assessment in M. Abscessus-infected ZF and the Use of Ziehl-Neelsen Staining

Dechorionated and anesthetized ZF embryos at 30 to 48 h post-fertilization (hpf) were injected with 3 nL of titrated *M. abscessus* expressing GFP frozen stock containing ~400 CFU into the caudal vein using a Nanoject III microinjector (Drummond Scientific, Broomall, PA, USA) as previously described [21]. The infection size (3 nL) was enumerated on 7H10^T/OADC^ supplemented with 50 μg/mL kanamycin. To follow in vivo efficacy of compounds and survival rate of infected embryos, infected larvae were transferred into 96-well plates (2 embryos/well), and incubated at 28.5 °C.

The in vivo efficacy of compounds was tested by adding directly into the fish water containing infected larvae in a 96-well plate at a final concentration of 25 µM; the infected embryos (without treatment) were used as a negative control. The fish water was renewed once daily. The drug efficacy of each compound was evaluated in three different ways after treatment; i) observation of *M. abscessus* CIP 104536^T^ R expressing GFP signal in ZF. The GFP development within the ZF was determined by observing the GFP evolution using a SteREO Lumar. V12 stereomicroscope with fluorescence optics (Zeiss, Jena, Germany) [24]. ii) measuring bacterial burden. To quantify the bacterial load at 5 days post infection (dpi), 3 infected embryos per group were collected and homogenized in 2% Triton X-100–PBST using a handheld homogenizer (D1000; Benchmark Scientific, Sayreville, NJ, USA). Serial 10-fold dilutions of the lysates were made and plated out on 7H10^T/OADC^ containing 50 µg/mL kanamycin and BBL Mycobacteria growth indicator tubes (MGIT) PANTA (polmyxin B, amphotericin B, nalidixic acid, trimethoprim, and azlocillin; Becton Dickinson, Franklin Lakes, NJ, USA). The plate was incubated for 3 to 5 days at 37 °C. iii) time-kill kinetics of *M. abscessus* infected ZF. Dead ZF (no heartbeat) were recorded daily up to 13 days to generate a survival curve. The survival curve was plotted by Prism using the method from Kaplan and Meier, with log-rank (Mantel–Cox) test.

For Ziehl–Neelsen staining, five fish were euthanatized at the indicated times. Fish were fixed for at least 96 h in 10% formalin and then dehydrated with ethanol. After paraffin embedding and sectioning, serial paraffin sections (5 μm) were prepared and subjected to modified Ziehl–Neelsen staining (BA-409 TB stain kit; Baso, Zhuhai, China) according to the manufacturer’s instructions. Sections were examined under an Olympus BH2 microscope (Tokyo, Japan), and images were recorded using a digital camera (TKC1481BEC; JVC, Tokyo, Japan).

## Figures and Tables

**Table 1:** Ninety percent minimum inhibitory concentration (MIC_90_) of rifamycin O against reference subspecies of the *M. abscessus* complex in comparison with reference compounds in 7H9^G/T/ADC^.

Strains	MIC_90_ (μM)				
	RFM O	RFB	RIF	RFT	CLR
*M. abscessus subsp. abscessus* CIP104536^T^	6.2	4.0	>50	>50	1.4
*M. abscessus subsp. bolletii* CIP108541^T^	4.0	7.1	>50	46.6	1.5
*M. abscessus subsp. massiliense* CIP108297^T^	5.6	4.5	47.3	>50	0.2

RFMO, rifamycin O; RFB, rifabutin; RIF, rifampicin; RFT, rifapentine; and CLR, clarithromycin.

## Supplementary Materials

The following are available online at https://www.mdpi.com/1420-3049/25/7/1597/s1, Figure S1. Cytotoxicity of RFM O against BMDMs. BMDMs were treated with the RFM O for 24 h. Cell death of BMDMs was analyzed by a Quanti-Max WST-8 Cell Viability assay kit (BIOMAX). Data are shown as the mean + S.D. of triplicate. TritonX-100 was used as a negative control and DMSO was used as a positive control. Figure S2. Toxicity evaluation using ZF. Kaplan-Meier larvae survival curves following treatment at 3 dpi with RFM O (A), RFB (B), and RIF(C) at concentrations of 5, 10, and 25 µM. The data were representative of three replicates, with 7 larvae per condition per replicate. Larvae were observed for survival for 12 days.
