# Supplementary figures and images for: Rifamycin O, An Alternative Anti-Mycobacterium abscessus Agent
Source: Molecules. 2020 Mar 31;25(7):1597. doi: 10.3390/molecules25071597 (PMC7181020; doi:10.3390/molecules25071597)

## Slide 1
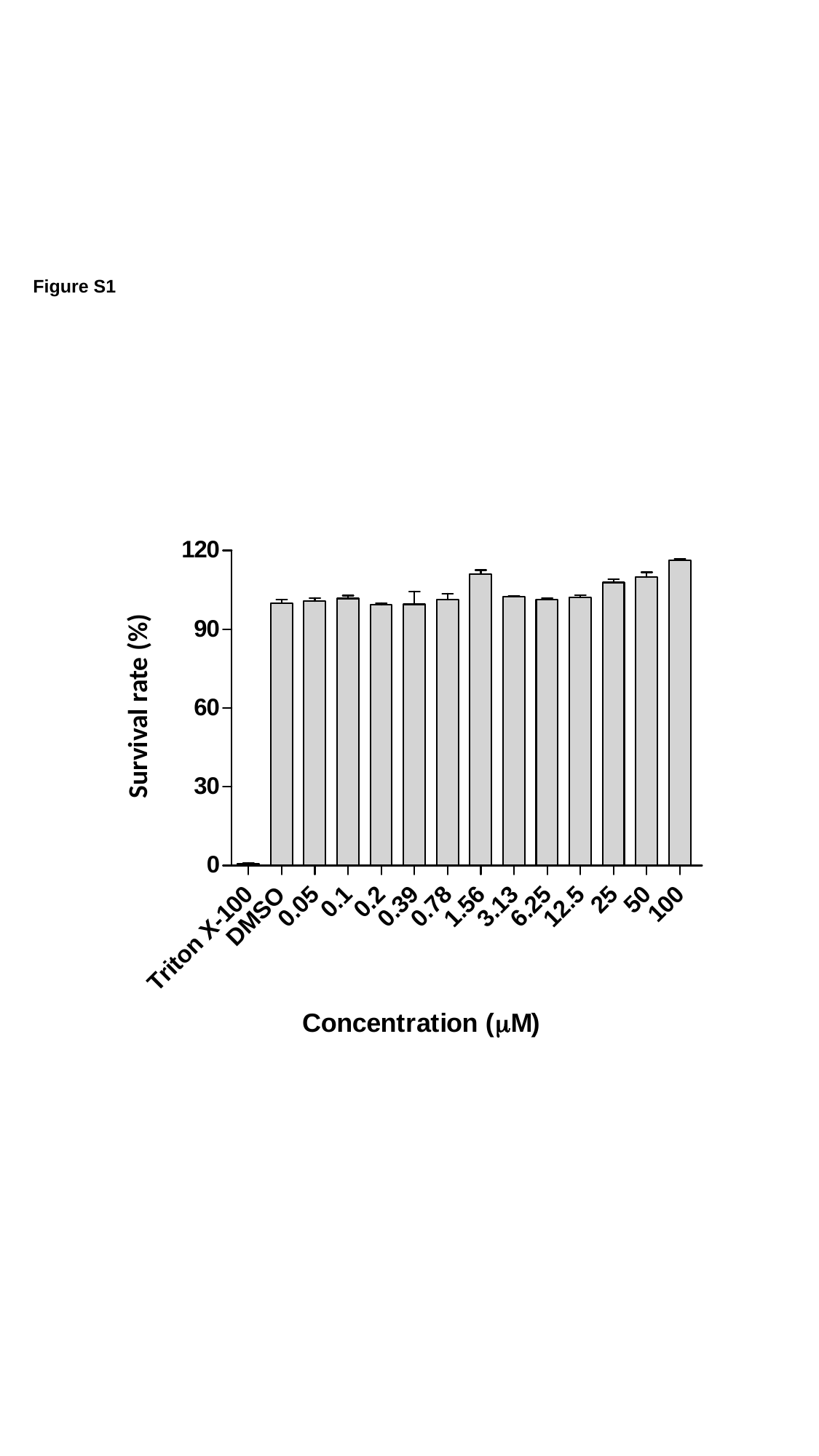

Figure S1
Survival rate (%)

## Slide 2
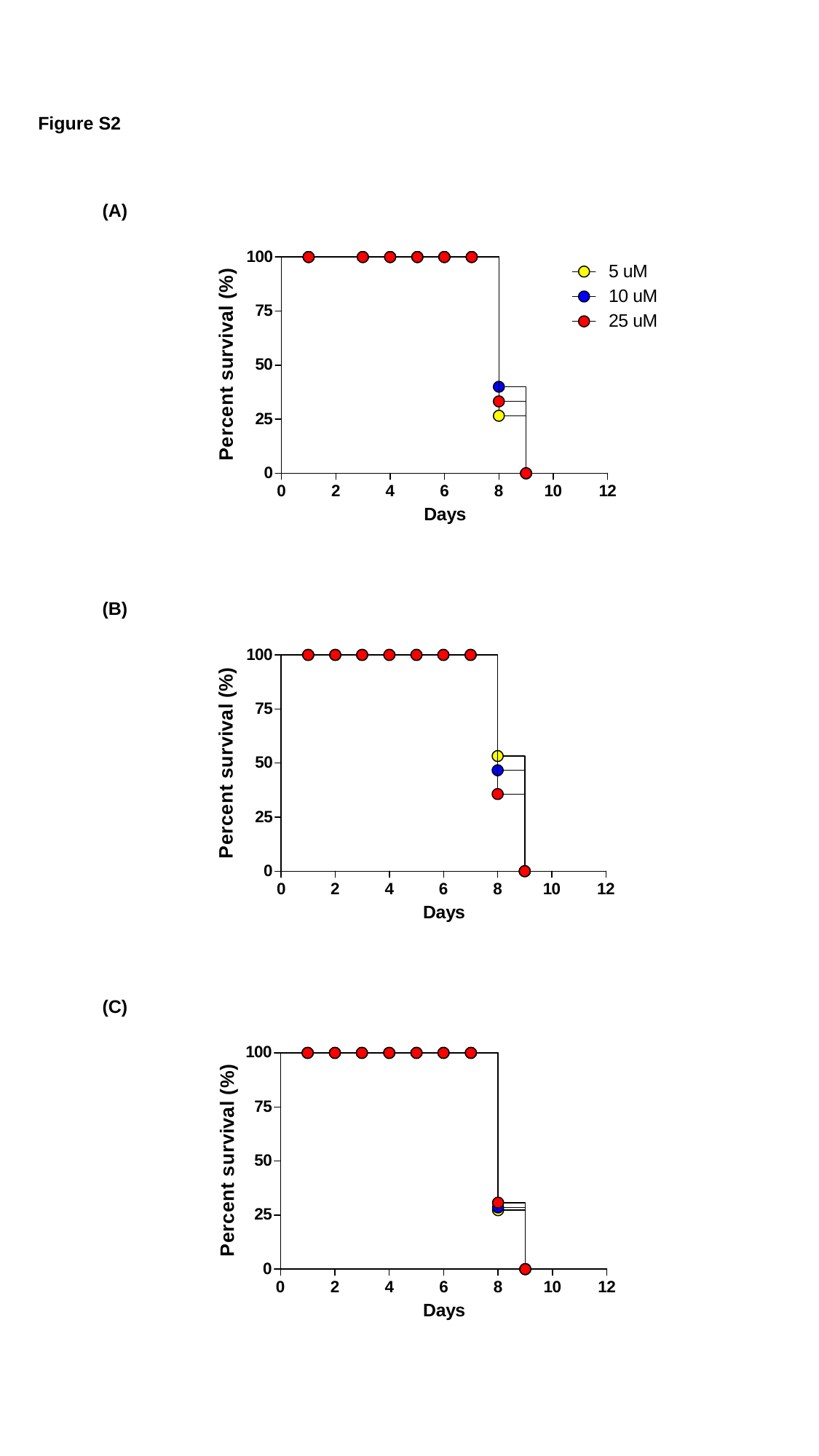

Figure S2
(A)
(B)
(C)

Supplement: Supplementary file 1 [file molecules-25-01597-s001.zip › molecules-724462-SI.pptx]
